# Food insecurity among people who inject drugs in Athens, Greece: a study in the context of ARISTOTLE programme

**DOI:** 10.1017/S1368980020004309

**Published:** 2021-04

**Authors:** Vana Sypsa, Eleni Flounzi, Sotirios Roussos, Angelos Hatzakis, Vassiliki Benetou

**Affiliations:** Department of Hygiene, Epidemiology and Medical Statistics, School of Medicine, National and Kapodistrian University of Athens, 75 Mikras Asias Street, 11527 Athens, Greece

**Keywords:** Food insecurity, Prevalence, HIV infection, Greece, ARISTOTLE programme

## Abstract

**Objective:**

To measure the prevalence of food insecurity and explore related characteristics and behaviours among people who inject drugs (PWID).

**Design:**

Cross-sectional analysis of a community-based programme for HIV infection among PWID (ARISTOTLE programme). Food insecurity was measured by the Household Food Insecurity Access Scale. Computer-assisted interviews and blood samples were also collected.

**Setting:**

A fixed location in Athens Metropolitan Area, Greece, during 2012–2013.

**Participants:**

In total, 2834 unique participants with history of injecting drug use in the past 12 months were recruited over four respondent-driven sampling rounds (approximately 1400/round).

**Results:**

More than 50 % of PWID were severely or moderately food insecure across all rounds. PWID were more likely to be severely food insecure if they were older than 40 years [adjusted OR (aOR): 1·71, 95 % CI: 1·33–2·19], were women (aOR: 1·49, 95 % CI: 1·17–1·89), from Middle East countries (aOR *v*. from Greece: 1·80, 95 % CI: 1·04–3·11), had a lower educational level (primary or secondary school *v*. higher education; aOR: 1·54, 95 % CI: 1·29–1·84), had no current health insurance (aOR: 1·45, 95 % CI: 1·21–1·73), were homeless (aOR: 17·1, 95 % CI: 12·3–23·8) or were living with another drug user (aOR: 1·55, 95 % CI: 1·26–1·91) as compared with those living alone or with family/friends. HIV-infected PWID were more likely to be severely food insecure compared with uninfected (59·0 % *v*. 51·0 %, respectively, *P* = 0·002); however, this difference was attributed to the confounding effect of homelessness.

**Conclusions:**

Moderate/severe food insecurity was a significant problem, reaching > 50 % in this sample of PWID and closely related to socio-demographic characteristics and especially homelessness.

Injecting drug use is a major contributor to the global burden of HIV and hepatitis C and B, through the usage of contaminated injecting equipment^(1)^. In 2015, approximately 15·6 million people, aged 15–64 years, injected drugs globally^(2)^. People who inject drugs (PWID) comprise a vulnerable population in high risk of poverty, unemployment, homelessness, incarceration and sex work, all of which are associated with increased blood-borne virus transmission^(2,3)^.

Food insecurity is an important social and public health problem, especially at times of declining public health spending and economic recession. Food insecurity, defined as having uncertain or limited availability of nutritionally adequate or safe food, or the inability to acquire personally acceptable foods in socially acceptable ways, leads to diminished physical and mental health status and negatively affects social and psychological well-being^(4)^. Food insecurity is more frequent among individuals with low income, lack of a stable shelter, unemployed or in social welfare, with a history of illegal drug use and mental health problems^(3)^.

Prevalence of food insecurity among PWID ranges between 30 % and 70 %, although evidence is rather limited^(3,5,6)^. Food insecurity may contribute to unsafe injection practices and interferes with access to health and social support programmes for PWID, thus increasing the risk of HIV transmission^(7)^. Food insecurity has also been associated with reduced antiretroviral treatment effectiveness and suboptimal treatment adherence^(8,9)^. On the other hand, HIV/AIDS can contribute to food insecurity through the debilitation of the most productive household members, the decrease of individual and household economic capacity and the increase of caregiver burden^(9)^. Data from resource-rich settings indicate a high prevalence of food insecurity among HIV-positive individuals^(10–12)^, as well as among HIV-HCV co-infected populations^(13,14)^.

The aim of the current study was to estimate the prevalence of food insecurity among PWID living in Athens Metropolitan Area in Greece and to explore its potential association with characteristics and behaviours of this population as well as HIV infection.

## Materials and methods

The ARISTOTLE programme is a ‘Seek-Test-Treat’ intervention programme, implemented during 2012–2013 among PWID in Athens, Greece, during an HIV outbreak in this population^(15)^. Details on the aim, study design, methodology and population characteristics have been described elsewhere^(16–18)^. In brief, ARISTOTLE was a community-based programme aiming to decrease HIV transmission among PWID by implementing a model of care that involved reaching out to high-risk, hard to reach PWID (‘seek’), engaging them in HIV testing (‘test’), and initiating HIV care, opioid substitution treatment and antiretroviral therapy (‘treat’). The programme has been implemented by the National and Kapodistrian University of Athens in collaboration with the Greek Organisation Against Drugs and approved by the Institutional Review Board of the Medical School of the University.

### Recruitment

Recruitment was performed using respondent-driven sampling (RDS). RDS begins with a limited number of initial recruits (seeds) and uses a coupon referral scheme that requests individuals to draw from their existing social networks to identify potential recruits^(19,20)^. ARISTOTLE was implemented in five consecutive RDS sampling rounds with a short break in between. Recruitment target was approximately 1400 PWID per sampling round. Each round lasted 10–12 weeks with 1 week to 1 month interval between the rounds. PWID could participate in multiple rounds, but only once in each round. Eligibility criteria were (a) presentation of a valid coupon, (b) injection of drugs without a prescription in the past 12 months, (c) residing in Athens metropolitan area, (d) age ≥ 18 years and (e) the ability to complete the interview in Greek or with the help of cultural mediators. As the questionnaire was introduced in the second sampling round, we analysed data from rounds 2 to 5. For PWID who participated in multiple rounds, we used their first visit with available food insecurity data.

### Measurement of food insecurity

The *Household Food Insecurity Access Scale (HFIAS)* a validated, widely used scale was implemented. The HFIAS was developed in the context of the US Agency for International Development’s Food and Nutrition Technical Assistance project and has been applied in numerous countries around the world, particularly in resource-constrained settings^(21)^. HFIAS consists of nine questions that cover three main domains of the experience of food insecurity: (a) anxiety and uncertainty about food supply, (b) insufficient quality and variety of food and (c) insufficient food intake and its consequences. Each of the nine questions ask whether a specific condition associated with the experience of food insecurity ever occurred during the previous 4 weeks and then how often this reported condition occurred during that period. The HFIAS was translated in Greek, administered and scored according to standard guidelines. The scale can take scores from 0 to 27, with higher scores reflecting more severe food insecurity. The respondents were assigned to one of the following four categories based on a specific categorisation scheme^(21)^ as follows: (1) food secure, (2) mildly food insecure, (3) moderately food insecure and (4) severely food insecure.

### Collection of other data

A computer-assisted interview, administered by trained personnel, was employed using a structured questionnaire with sections on socio-demographic characteristics, injection and sexual behaviours, access to prevention and treatment^(16)^. Blood samples were also collected, and HIV tests were performed with a microparticle EIA anti-HIV-1/2 (AxSYM HIV-1/2 gO, Abbott). HIV-1 and HIV-2 infection was confirmed by Western Blot (MP Diagnostics).

### Statistical analysis

Descriptive statistics estimating median, 25th and 75th percentiles of food insecurity scores were obtained for each sampling round. Wilcoxon rank-sum test was used to assess whether there was a statistically significant change in the scores of the second and the fifth round (December 2012–March 2013 *v*. September–December 2013). We obtained crude (sample) as well as RDS-weighted (population) estimates for the prevalence of the various levels of food insecurity (food secure, mild, moderate, severe food insecurity) by sampling round with RDS Analysis Tool (RDSAT)^(22)^.

We further explored which factors were associated with the presence of severe food insecurity among PWID. Crude and adjusted OR of severe food insecurity along with 95 % CI were obtained using logistic regression. In multivariable analysis, we accounted for correlation between recruiter and recruits due to the RDS sampling using generalised estimating equations logistic regression. We created a variable indicating who the recruiter of each subject was and used this as a cluster variable in the generalised estimating equations algorithm. An exchangeable correlation structure within each cluster was assumed. Multivariable analysis also accounted for differential recruitment effectiveness by HIV status and for the differing sample inclusion probabilities. We calculated inverse probability weights based on individualised recruitment weights and included them as covariate in the logistic regression models. These weights combine the individualised degree component (the inverse of the network size of the participant) with an adjustment for differential recruitment^(23),^ and were derived via RDSAT. We also adjusted for the different rounds of the programme by including an indicator variable in the model. Covariates included age (three categories: 18–30 years, 31–40 years and > 40 years), gender (female *v*. male), country of origin (Greek, Iran/Iraq/Afghanistan/Pakistan and other), education (high school or higher *v*. primary school and middle/secondary school together), current living status (living without a drug user, living with a drug user and homeless), current health insurance (yes *v*. no), main substance use (Heroin/Thai, Cocaine, Speedball and Other), anti-HIV status (positive *v*. negative), use of drugs divided with a syringe that someone else had already injected with in past 12 months (never, rarely/about half the time and most of the time/always) and round of ARISTOTLE programme.

## Results

Data on HFIAS were available for ARISTOTLE participants from the second until the fifth sampling round, and more specifically, from 1429 PWID in the second round, 1428 in the third round, 1407 in the fourth round and 1397 in fifth round. Over the four sampling rounds, 2834 unique participants (men and women) were recruited.

Table 1 presents the median, 25th and 75th percentile of the food insecurity score in each sampling round, as well as the distribution of participants in each category of the food insecurity scale. Across all rounds, more than 50 % of PWID were severely or moderately food insecure, whereas < 1/3 (37 %–41 % per round) were food secure. An improvement was observed in the median total food insecurity score in the 5th round as compared with the second round (7 *v*. 10, *P* < 0·001).

**Table 1:** Food insecurity score (median, 25th and 75th percentile) and distribution (numbers and percentage) of people who inject drugs (PWID) in each category of the food insecurity scale by sampling round: the ARISTOTLE programme

	2nd round		3rd round		4th round		5th round	
Food insecurity scale	*n*	%	*n*	%	*n*	%	*n*	%
Number of participants	1429		1428		1407		1397	
Time period	December 2012–March 2013		March–June 2013		June–September 2013		September–December 2013	
Median score	10		9·5		9		7	
25th, 75th percentile	0, 19		0, 18		0, 18		0, 18	
Severely food insecure category^*^	777	46·9	694	41·7	691	41·4	627	43·5
Moderately food insecure category^*^	144	12·0	155	9·9	139	13·3	143	10·5
Mildly food insecure category^*^	57	4·5	109	7·7	97	4·2	109	4·9
Food secure category^*^	451	36·6	470	40·6	480	41·1	518	41·1

* RDS-weighted proportions.

Based on the data collected in all rounds of first participation, the highest proportion of severe food insecurity was identified among PWID from Iran/Iraq/Afghanistan/ Pakistan (81·3 %) and homeless (92·7 %) as presented in Table 2.

**Table 2:** Severe food insecurity according to selected characteristics for people who inject drugs (PWID) at their first participation to ARISTOTLE programme (*n* = 2834) along with crude and adjusted odds ratios (OR) and 95 % confidence intervals (95 % CI) for the risk of being severely insecure

Baseline characteristics	Severe food insecurity		Crude OR	95 % CI	Adjusted OR^*^	95 % CI
	*n*	%				
Age (years)						
18–30	338	51·2	1·00		1·00	
31–40	678	48·2	0·88	0·74, 1·07	1·11	0·89, 1·38
>40	459	60·5	1·46	1·18, 1·80	1·71	1·33, 2·19
Gender						
Men	1204	51·0	1·00		1·00	
Women	271	58·3	1·34	1·10, 1·64	1·49	1·17, 1·89
Country of origin						
Greece	1201	49·6	1·00		1·00	
Iran/Iraq/Afghanistan/Pakistan	100	81·3	4·43	2·79, 7·01	1·80	1·04, 3·11
Other	174	62·1	1·67	1·30, 2·16	1·00	0·73, 1·37
Education						
Primary school, Middle/Secondary school	940	58·4	1·78	1·53, 2·07	1·54	1·29, 1·84
High School or higher	526	44·1	1·00		1·00	
Current living status						
Living alone or with family/friends	602	36·9	1·00		1·00	
Living with a drug user	287	51·2	1·79	1·48, 2·18	1·55	1·26, 1·91
Homeless	581	92·7	21·6	15·8, 29·7	17·1	12·3, 23·8
Currently health insurance						
Yes	430	40·4	1·00		1·00	
No	1042	59·3	2·15	1·84, 2·51	1·45	1·21, 1·73
Main substance of use						
Heroin/Thai†	1220	54·8	1·73	1·41, 2·13	1·44	1·14, 1·82
Cocaine	185	41·2	1·00		1·00	
Speedball	49	45·4	1·19	0·78, 1·81	1·15	0·70, 1·90
Other	17	51·5	1·52	0·75, 3·08	1·93	0·90, 4·16
Anti-HIV						
Negative	1230	51·0	1·00		1·00	
Positive	245	59·0	1·38	1·12, 1·71	0·98	0·76, 1·28
Use drugs divided with a syringe that someone else had already injected with (past 12 months)						
Never	905	49·0	1·00		1·00	
Rarely, About half the time	531	57·9	1·43	1·22, 1·68	1·28	1·06, 1·55
Most of the time, Always	36	65·5	1·98	1·13, 3·47	1·64	0·85, 3·20
Round						
round 2 (Dec2012–Mar2013)	787	54·7	1·00		1·00	
round 3 (Mar–Jun2013)	297	49·0	0·80	0·66, 0·96	0·77	0·61, 0·96
round 4 (Jun–Sep2013)	224	51·6	0·88	0·71, 1·10	0·83	0·65, 1·07
round 5 (Sep–Dec2013)	167	48·0	0·76	0·60, 0·97	0·67	0·51, 0·89

* Accounting for correlation between recruiter and recruits, differential recruitment effectiveness by HIV status and differing sample inclusion probabilities.

† Thai: mix of cheap heroin and sedatives.

Based on univariable analysis (Table 2), higher risk of severe food insecurity was identified among PWID aged >40 years, women, from Iran/Iraq/Afghanistan/Pakistan or other countries, with low educational level, living with drug users or homeless (as compared with PWID living alone or with family/friends), with heroin as main use of substance, with HIV infection, as well as those who used drugs divided with a used syringe. HIV-infected PWID were more likely to be severely food insecure as compared with uninfected PWID (59·0 % *v*. 51·0 %, respectively, *P* = 0·002).

In the multivariable analysis, PWID had higher risk of severe food insecurity if they were > 40 years [compared with 18–30 years; adjusted OR (aOR): 1·71, 95 % CI: 1·33–2·19], were women [aOR: 1·49, 95 % CI:1·17–1·89], from Iran/Iraq/Afghanistan/Pakistan compared with PWID from Greece [aOR: 1·80, 95 % CI: 1·04–3·11], had a lower educational level (primary or secondary school *v*. higher education; aOR: 1·54, 95 % CI: 1·29–1·84), were living with another drug user (aOR: 1·55, 95 % CI: 1·26–1·91) or were homeless (aOR: 17·1, 95 % CI: 12·3–23·8), compared with living alone or with family/friends and had no current health insurance (yes *v*. no insurance; aOR: 1·45, 95 % CI: 1·21–1·73) (Table 2). Use of drugs divided with a syringe that someone else had already injected with rarely or about half of the times (*v*. never) in the past 12 months and use of heroin as main substance use (*v*. cocaine) were both associated with higher risk of being severely insecure. HIV status was no longer statistically significant. The association identified in the univariable analysis was mainly attributed to the confounding effect of homelessness; 35·9 % of HIV-infected individuals were homeless as compared with 19·8 % of uninfected.

The improvement in food insecurity across rounds was confirmed in the multivariable analysis; compared with the participants of the second round (December 2012–March 2013), participants of the third and fifth rounds (March–June 2013 and Sep–Dec 2013, respectively) had 23 % and 33 % lower risk of being severely food insecure (OR [95 % CI]: 0·77 [0·61–0·96] and 0·67 [0·51–0·89], respectively).

## Discussion

In this sample of PWID living in Athens, Greece, the prevalence of moderate or severe food insecurity was high and exceeded 50 %. Being a female, older than 40 years, homeless or living with a drug user, from Middle East countries, having a lower educational level and no health insurance coverage were all associated with higher odds of being severely food insecure. Levels of severe food insecurity were higher among HIV-positive PWID compared with HIV-negative ones, but after adjusting for homelessness, HIV status was not associated with severe food insecurity.

Our findings are in accordance with previous studies reporting high levels of food insecurity among PWID in economically developed countries^(5,6)^. In a cross-sectional study conducted among 777 PWID from Los Angeles and San Francisco, USA, over half of the participants (58 %) reported food insecurity (measured by the US Adult Food Security Survey ten-item Module), while 41 % met USDA criteria for very low food security status^(6)^. Food insecurity was associated with being homeless, engaging in distributive syringe sharing and feeling at risk for arrest for possession of drug paraphernalia but not with age, gender, ethnicity or race. In the context of the Vancouver Injection Drug Users Study, conducted among HIV-negative PWID, 64·7 % of the participants experienced hunger and were unable to afford enough food^(5)^.

Women were more likely to be food insecure compared with men. Gender inequalities have been reported by other studies also in which women were the most affected individuals maybe because of gender discrimination, poverty and lack of support as head of a household^(24)^.

Lower education, lack of health insurance and homelessness, all indicators of lower socio-economic status, were associated with higher food insecurity risk. Of note is the particularly high risk of food insecurity among homeless PWID. Serious limitation of homeless individuals to store and preserve food, prepare proper meals and their associated stress have been proposed to explain this association^(5,10,11)^. In other studies, severe food insecurity was positively associated with current living status and particularly with homelessness and living with a drug user, which both have been associated with increased probability of acquiring or transmitting HIV^(3,6,7)^ and may partly explain the close link observed between food insecurity and HIV in other populations^(4,25,26)^. In our population, severe food insecurity was more prevalent among HIV-infected individuals, mainly due to the fact that they were more likely to be homeless. This finding has serious implications among people living with HIV since food insecurity has been associated with non-adherence to antiretroviral treatment as well as reduced antiretroviral treatment effectiveness^(8,9,27)^. PWID reporting sharing a syringe rarely to about half of the times had 28 % higher odds of being severe food insecure as compared with those who never practiced this behaviour (OR: 1·28 ; 95 % CI: 1·06–1·55). This difference was not significant for PWID that always shared the syringe compared with those who never, in the multivariable analysis, most probably due to the small number of people reporting always dividing drugs with a used syringe. This association has also been reported before^(3,10)^.

The proposal of a conceptual framework that could elucidate the links between food insecurity and injecting drug use would be useful. The framework proposed by Weiser and colleagues for food insecurity and HIV/AIDS^(28)^ provides some further insight, since similarities with food insecurity among PWID are evident. Thus, socio-economic factors at the community and household level, such as poverty, lack of education or access to information, as well as social factors, such as gender inequalities, drug-user related stigma and lack of social support, may influence food insecurity among PWID.

Among the limitations of our study is the cross-sectional design, which does not allow drawing conclusions upon causality of the identified associations. The questionnaire used for recording food insecurity measures access to food which is only one of the dimensions of food insecurity, thus not fully capturing the extent and magnitude of food security^(29)^. Also, it has not been formally validated in the Greek population, although it is widely used in international environments. Among the strengths of the current study is that, to our knowledge, it is the first one that investigates food insecurity and its risk factors among PWID in Greece, recruited through a community-based program during an ongoing HIV outbreak. Furthermore, although access to PWID is very difficult and almost precludes a random sampling, the population coverage of the ARISTOTLE programme is estimated to be high, reaching 72 % (based on the number of participants in rounds 2–5 and the official capture–recapture estimate for the size of the PWID population in Athens), allowing us to claim that our findings represent a large portion of the specific population^(17)^. More affluent PWID are probably less likely to participate to a similar programme; however, RDS makes it possible to identify a hard to reach population in the absence of a sampling frame and in particular the population most in need (active PWID with no access to services).

In conclusion, severe and moderate food insecurity was recorded in more than 50 % of the population of PWID participating in the current study, while several socio-demographic and other characteristics were associated with its existence and severity. Although there are no available data on food insecurity in more recent years in this population, homelessness in a sample of PWID recruited using the same methodology in 2018–2019 remains as high as in 2012–2013 (27·2 % *v*. 23·1 %, respectively)^(16,30)^. In addition, services providing food to homeless people (including PWID) were affected during the COVID-19 pandemic when stringent social distancing measures were implemented. As food insecurity seems to consist a substantial problem among PWID, implementation of focused strategies in order to improve food access and housing of PWID, especially among women, should be among the priorities of an effective health policy planning.
